# Mechanisms of Ovarian Cancer Metastasis: Biochemical Pathways

**DOI:** 10.3390/ijms130911705

**Published:** 2012-09-18

**Authors:** Kentaro Nakayama, Naomi Nakayama, Hiroshi Katagiri, Kohji Miyazaki

**Affiliations:** 1 Department of Obstetrics and Gynecology, Shimane University School of Medicine, Izumo 693-8501, Japan; E-Mails: jimipin999_lessismore@yahoo.co.jp (H.K.); kennaonatsuno@docomo.ne.jp (K.M.); 2 Department of Biochemistry, Shimane University School of Medicine, Izumo 693-8501, Japan; E-Mail: nn0411@med.shimane-u.ac.jp

**Keywords:** cancer, metastasis suppressor gene, EMT, tumor microenvironment

## Abstract

Ovarian cancer is the most lethal gynecologic malignancy. Despite advances in chemotherapy, the five-year survival rate of advanced ovarian cancer patients with peritoneal metastasis remains around 30%. The most significant prognostic factor is stage, and most patients present at an advanced stage with peritoneal dissemination. There is often no clearly identifiable precursor lesion; therefore, the events leading to metastatic disease are poorly understood. This article reviews metastatic suppressor genes, the epithelial-mesenchymal transition (EMT), and the tumor microenvironment as they relate to ovarian cancer metastasis. Additionally, novel chemotherapeutic agents targeting the metastasis-related biochemical pathways are discussed.

## 1. Introduction

Malignancy is one of the most common causes of death, with most patients dying of complications of metastatic disease. Tumor cell metastasis is the culmination of a multi-step process including (1) Disconnection of intercellular adhesions and separation of single cells from solid tumor, (2) Invasion of lymphatic and blood vessels, (3) Immunologic escape in the circulation, (4) Adhesion to endothelial cells, (5) Extravasation from lymph-and blood vessels and (6) Proliferation and induction of angiogenesis.

Ovarian cancer is more likely to metastasize via intraperitoneal dissemination than hematogenously or via lymphatics. Intraperitoneal spread appears to be an early event, which is why these cancers are only rarely detected at an early stage. Growth factors and cytokines within the tumor microenvironment induce the epithelial-mesenchymal transition (EMT), through which tumor cells acquire motility and invasion ability. Recent evidence suggests that tumor stem cells likely participate in the EMT. The complex biochemical pathways involved in this process, however, have only just begun to be elucidated.

In this issue, we begin by addressing the canonical metastasis suppressor genes and transition to discussing the biochemical and cellular processes in ovarian cancer metastasis including the EMT, the tumor microenvironment, anoikis resistance, microRNA, and chemokines.

## 2. Metastasis Suppressor Genes

Metastasis represents the culmination of a complicated series of events that includes the separation of single cells from a solid tumor, venous invasion, immunologic escape in the circulation, adhesion to endothelial cells, extravasation from lymph- and blood vessels, proliferation, and the induction of angiogenesis. The completion of these steps requires the interaction of multiple signaling pathways.

Presently, approximately 30 canonical metastasis suppressor genes have been identified in various cancers. They contribute to carcinogenesis indirectly through loss of function. They frequently encode proteins related to cell-cell adhesion, apoptosis, and cell invasion, and frequently suppress EMT induction [1]. Those genes that have been identified in the context of ovarian cancer metastasis are summarized in Table 1 [1,2].

### 2.1. Nm23

The first metastasis suppressor gene, Nm23, was identified by a complimentary DNA (cDNA) subtraction approach [15]. Nm23 is a strong inhibitor of tumor migration. This migration suppressive role of Nm23 is not mediated by its NDP kinase activity and appears to specifically affect directional cell invasion [16,17]. Nm23 expression is inversely correlated with poor survival and tumor grade for ovarian carcinomas [3].

### 2.2. KISS1 and KISS1R

KISS1 is a secreted protein that is processed into polypeptides termed kisspeptins by prohormone convertases such as furin [18,19]. KISS1 and certain kisspeptins such as KP54 are able to bind the KISS1 receptor (KISS1R) and inhibit the formation of metastases *in vivo* [4,19]. KISS1R binds internal fragments derived from KISS1, one of the first described metastasis suppressors. Initially, this interaction was thought to form an autocrine loop by which the tumor cells suppressed metastasis. Kisspeptin and GPR54 immunoreactivity are significantly associated with favorable prognosis in both disease specific and overall survival. They are significantly associated with the clear cell ovarian carcinoma subtype, thereby creating the first independent prognostic biomarkers specific for ovarian clear cell carcinomas [4,5].

### 2.3. KAI1

*KAI1* is a member of the tetraspanin or transmembrane 4 superfamily (TM4SF). These type III transmembrane proteins encompass cytoplasmic *N*- and *C*-termini, and traverse the cell membrane four times, thereby forming one small and one large extracellular loop. Tetraspanins are implicated in the regulation of cell motility, morphology, fusion, signaling, fertilization, and differentiation. By these cellular activities, some of them are also involved in the regulation of certain pathologies, including cancer metastasis. The tetraspanin KAI1 was originally identified as a suppressor of metastatic spread in a rat prostate cancer model [20]. Its loss or low expression directly correlates with poor prognosis in ovarian cancer [6]. KAI1 may suppress ovarian cancer progression by inhibiting integrin αvβ3/vitronectin-provoked tumor cell motility and proliferation [7].

### 2.4. E-Cadherin

In many tumor types, *E*-cadherin loss occurs during the epithelial-to-mesenchymal transition (EMT) [21], which correlates with invasion and metastasis. Collectively, loss of E-cadherin is thought to be causative for invasion. E-cadherin expression can be regulated at transcriptional and posttranscriptional levels. In particular, the zinc finger transcriptional repressors Snail and Slug have been implicated in repressing E-cadherin transcription. Loss of E-cadherin correlates with shorter overall survival in ovarian cancer [8,9].

### 2.5. OGR1

Ovarian cancer G-protein coupled receptor (OGR1) is another GPCR metastasis suppressor in prostate carcinoma cells [22]. OGR1 regulates endothelial barrier integrity, proliferation and tube formation, and T-cell migration. There is also data suggesting that OGR1 and related family members have proton sensing properties.

Over-expression of OGR1 in human ovarian cancer cells significantly inhibits cell proliferation and migration, and results in enhanced cell adhesion to the extracellular matrix. Therefore, OGR1 may be a metastatic suppressor in ovarian cancer [10]. Since it is expressed on the cell surface, it is presumed that OGR1 is involved in receiving or transmitting signals from the tumor cell; however, the nature of the signal(s) is not definitively known.

### 2.6. BRMS1

Breast cancer metastasis suppressor 1 (BRMS1) is a predominantly nuclear protein that differentially regulates the expression of multiple genes. BRMS1 acts to suppress metastasis without affecting orthotopic tumor growth. Ectopic expression of BRMS1 in highly metastatic ovarian cancer cells suppresses metastasis by impairing colony formation [11]. Recently, Sheng *et al*. reported that BRMS1 might regulate the metastatic potential at least in part through upregulation of CXCR4 via NF-κB activation in ovarian cancer [12].

## 3. EMT (Epithelial-Mesenchymal Transition)

In order to metastasize to distant organs, cancer cells must first detach from neighboring cells. Epithelial cells are connected by a specialized adhesion complex and have apical-basal polarity. In contrast, mesenchymal cells lose these junctions and transition into motile, spindle-shaped cells with front-to-back cell polarity. Epithelial cells can convert to mesenchymal cells via a multi-step process referred to as the epithelial-mesenchymal transition (EMT), which is characterized by dramatic phenotypic changes. The hybrid cell co-expresses both epithelial and mesenchymal traits and is considered to have a phenotype capable of metastasis.

The epithelial-mesenchymal transition (EMT) was originally described by Hay *et al.* in 1980. It was subsequently shown to be critical in many developmental processes. For example, the formation of separate tissue layers from a single epithelial layer during gastrulation is associated with the EMT.

There is strong evidence that the EMT also plays a central role in tumor progression. During progression to metastatic competence, cancer cells acquire mesenchymal properties. This results in changed adhesive properties and activation of motility, which allow tumor cells to metastasize and establish secondary tumors at distant sites.

EMT changes have recently been classified into three categories: type 1, type 2, and type 3 [23]. Type 1 occurs during embryogenesis. Type 2 occurs in the context of inflammation and tissue damage as part of the repair and remodeling processes. Finally, Type 3 EMT is involved in tumor progression [23].

E-cadherin, a marker of the epithelial phenotype, is an important metastasis suppressor gene. Downregulation of E-cadherin has several important consequences that are of direct relevance to the EMT. Therefore, loss of E-cadherin is a marker of the EMT. Several transcriptional factors which repress transcription of E-cadherin have been identified. These include Twist, Snail, and Slug, the downregulation of which induces the EMT. It has been reported that expression of these factors plays a central role in suppressing ovarian cancer metastasis [13,24].

Recently, we discovered that one of the metastasis suppressor genes, *MKK4*, was homozygously deleted in some ovarian cancer samples [14]. Loss of MKK4 resulted in the induction of Twist expression via phosphorylation of NF-κβ and repression of E-cadherin expression. Consequently, cancer cells acquire the EMT phenotype and gain the ability to metastasize [13].

We have recently shown that another cancer-related transcriptional factor, *NAC1*, also might be related to the EMT. *NAC1* negatively regulates *MKK4* via the GADD45 pathway [25]. When ectopically expressed in ovarian cancer cells, NAC1 increases cellular motility and invasive ability. Subsequent *NAC1* knockdown by siRNA in the same ovarian cancer cells reverses this effect [26]. NAC1 inhibitors therefore may not only induce cell death but also suppress the metastasis of ovarian cancer cells. To this effect, we are presently researching agents that inhibit NAC1 [27].

## 4. Resistance against Apoptosis and Anoikis

In normal tissue, epithelial cells maintain close ties to adjacent cells and the ECM (extracellular matrix). Communication between neighboring cells and the ECM provides essential signals for growth and survival. Normal cells enter apoptosis when separated from the ECM and neighboring cells (a process known as anoikis) [28]. When cells are detached from the ECM, there is a loss of the normal cell–matrix interaction, which often induces the cell to undergo anoikis, a form of programmed cell death. To successfully metastasize, tumor cells must suppress the initiation of anoikis [29,30]. In ovarian cancer cells, overexpression of RAB25, a member of the small GTPase family, confers resistant to anoikis [29]. Recently, Sood *et al*. reported that norepinephrine and epinephrine protect ovarian cancer cells from anoikis via a FAK-mediated signaling pathway that is initiated by ADRB2 and involves subsequent Src-associated phosphorylation of FAK^Y397^ [31].

## 5. Abnormal Expression of microRNA and Cancer Metastasis

The discovery of microRNAs (miRNAs) opened a new avenue for cancer metastasis research. It has become clear that alterations of non-coding genes, including miRNAs, also contribute to cancer pathogenesis. miRNAs control a wide range of physiological and pathological processes, including development, differentiation, cellular proliferation, programmed cell death, cancer initiation and metastasis, by modulating the expression of their cognate target genes through the cleavage of mRNA molecules or by inhibiting their translation. Importantly, a single miRNA can influence the expression of hundreds of proteins [32]. Some miRNAs can trigger feedforward or feedback loops by synergistically cooperating with their target genes [33]. These findings reveal an amazing network encompassing numerous molecules, many of which interact in novel ways. MicroRNAs (miRNAs) are an evolutionarily conserved group of small RNAs (18–24 nucleotides) that inhibit gene expression. miRNAs coordinate the expression of entire sets of genes, shaping the mammalian transcriptome. A large body of evidence suggests that the multigene regulatory capacity of miRNAs is dysregulated and exploited in cancer. Although the oncogenic and tumor-suppressive roles of some miRNAs in ovarian cancer have been determined by numerous studies, the specific action of miRNAs in mediating metastasis has been addressed only recently [34]. Explorations by *in vitro* screens for steps in the metastatic cascade have disclosed a molecular link that connects miRNA expression and metastasis. In breast cancer, miRNAs have been identified in metastatic tumor tissues and lymph nodes. miRNAs likely enhance metastasis by influencing multiple signaling pathways and targeting proteins critical in the different steps of metastasis. In ovarian cancer cell lines, certain miRNAs have been associated with the invasive and metastatic phenotype.The miR-200 family contributes to metastasis in ovarian cancer by targeting ZEB1 and ZEB2m, which in turn activate the transcription of E-cadherin [34]. miR373 and mir-520c are also dysregulated and foster metastasis in ovarian cancer [35].

Recently, Mateescu *et al*. identified crosstalk between oxidative stress and the miR-200 family of microRNAs that affects tumorigenesis and chemosensitivity [36]. miR-141 and miR-200a target p38α and modulate the oxidative stress response. Enhanced expression of these microRNAs mimics p38α deficiency and increases tumor growth and improves the response to chemotherapeutic agents in a mouse model. High-grade human ovarian adenocarcinomas that accumulate miR-200a have low concentrations of p38α and an associated oxidative stress signature. The miR200a-dependent stress signature correlates with improved survival of patients in response to treatment. Therefore, the role of miR-200a in stress could be a predictive marker for clinical outcome in ovarian cancer.

## 6. Tumor Microenvironment

The tumor microenvironment comprises a variety of nonmalignant stromal cells that play a pivotal role in tumor progression and metastasis. These stromal cells include inflammatory cells, immune cells, and hematopoietic cells.

Cancer-associated fibroblasts (CAF) are an important stromal constituent. CAF promote cancer progression and may represent a therapeutic target [37]. Co-culturing of ovarian cancer cells with CAF enhances the proliferation potency of the cancer cells and increases their metastatic capability *in vivo* [37].

CAF produce growth factors, such as IGF and HGF, and directly interact with cancer cells to promote growth and survival. TGF-β and HGF induce the EMT in cancer cells and promote invasion and metastasis. In ovarian cancer, TGF-β signaling, which is activated by CAF present in the omentum, contributes to metastasis [38]. CAF also contribute to the formation of the cancer stem cell niche through the production of chemokines [39].

There is strong evidence that mesenchymal stem cells (MSCs) are recruited to the tumor microenvironment. Based on this tropism of MSCs for the tumor microenvironment, numerous studies have suggested that MSCs could potentially be used as therapeutic vectors to target the tumor [40]. Carcinoma-associated MSCs (CA-MSCs) have recently been identified in ovarian tumor specimens [41]. These CA-MSCs have a normal morphologic appearance, a normal karyotype, and are non-tumorigenic. CA-MSCs are multipotent and have the capacity for differentiating into adipose tissue, cartilage, and bone. When combined with tumor cells *in vivo*, CA-MSCs promote tumor growth more effectively than MSCs. *In vitro* and *in vivo* studies suggest that CA-MSCs promote tumor growth by increasing the number of cancer stem cells [41].

Intra-abdominal tumors, such as ovarian cancer [42], have a clear predilection for metastasis to the omentum, an organ primarily composed of adipocytes. Currently, it is unclear why tumor cells preferentially home to, and proliferate in, the omentum; yet omental metastases typically represent the largest tumor in women with ovarian cancer. Primary human omental adipocytes promote homing, migration, and invasion of ovarian cancer cells. Adipokines, including interleukin-8 (IL-8), mediate these activities [43]. Fatty acid-binding protein 4 is upregulated in omental metastases as compared to primary ovarian tumors, and FABP4 expression is present in ovarian cancer cells at the adipocyte-tumor cell interface. FABP4 deficiency substantially impairs metastatic tumor growth in mice, indicating that FABP4 has a key role in ovarian cancer metastasis. It is therefore plausible that adipocytes provide fatty acids for rapid tumor growth. Lipid metabolism and transport, therefore, represent novel therapeutic targets for cancers in which adipocytes are a major component of the microenvironment.

Chemokines are small peptides that are potent activators and chemoattractants for leukocytes and other inflammatory cells. Chemotactic cytokines belong to the chemokine superfamily, which can be divided into four groups (CXCL, CCL, CL, and CX3CL), according to the positioning of the first two closely-paired and highly conserved cysteines of the amino acid sequence. The specific effects of chemokines on their target cells are mediated by members of a family of 7-transmembrane-spanning, G-protein-coupled receptors. Chemokines and their receptors have been implicated in tumor growth and tumor cell invasion. In particular, chemokine receptors elicit cancer cell mobilization and promote distant metastasis with a certain degree of organ selectivity. CXCR4 is one of the most frequently expressed chemokine receptors in ovarian cancer. A recent study highlighted the role of CXCR4 in promoting peritoneal metastasis and associated it with poor prognosis [44]. CAF express CXCL12, one of the cancer related chemokines [45]. This CAF-derived cytokine promotes tumor progression and metastasis. Recently, TCGA has shown that ovarian serous high grade carcinomas may be subdivided into one of four different groups based on cluster gene content [46]. In this report, metastasis-related chemokine receptor CXCR3 was overexpressed in ovarian serous high grade carcinomas [46].

Independent studies have demonstrated that in the ovarian carcinoma microenvironment, T cells (exclusively) spontaneously exert clinically relevant pressure against tumor progression [47,48]. In a tumor model using an immunocompetent host, anti-tumor immunity is also identified very early. This initial immunity is driven by infiltrating dendritic cells (DCs) that suppress tumor growth for a prolonged period [49]. Rapid tumor progression coincides with a phenotypic switch in the expanding DC infiltrates, with concomitant abrogation of T cell antitumor activity rather than a loss in tumor cell immunogenicity. An interesting observation is that depletion of DCs early in the disease course accelerates tumor expansion; but DC depletion at advanced stages significantly delays aggressive malignant progression. This suggests that DC may differ in their phenotype, with some recruited for immunosurveillance and others responsible for accelerated malignant growth.

As a result of rapid tumor growth, the tumor microenvironment is frequently hypoxic. HIF-1 (hypoxia inducing factor-1) is induced and functions as a transcription factor in the setting of tumor hypoxia. Several studies have addressed the relationship between ovarian cancer prognosis and *HIF-1* expression [50,51] with some studies associating the overexpression of *HIF-1* with a worse prognosis. HIF1 induces expression of Snail, which plays a central role in EMT induction in a hypoxic environment [52]. Hypoxia caused by tumor growth also induces EMT and contributes to tumor invasion and metastasis. In ovarian cancer, cancer cells produce CXCL12 and VEGF in response to hypoxia. As a result, they affect endothelial cells and induce tumor angiogenesis [53]. The importance of these processes in ovarian cancer metastasis, however, is debated, given that the most common route of spread is intraperitoneal seeding [54].

The CD44 glycoprotein is an acidic molecule whose charge is largely determined by sialic acid. CD44 is a multi-structural cell surface receptor which binds HA (hyaluronic acid) with a particularly high affinity [55]. It belongs to a family of transmembrane glycoproteins, which contain a variable extracellular domain, a 23-amino acid transmembrane domain, and a 70-amino acid cytoplasmic domain. Ovarian cancer cell adhesion to mesothelial cell monolayers is mediated at least in part by the interaction between HA and CD44 [56]. It has also been suggested that ovarian cancer cell interactions with mesothelial cell HA may also mediate tumor metastasis [57]. The addition of anti-CD44 antibodies has been reported to significantly decrease adhesion of ovarian cancer cells to HA [56]. *In vivo* studies have suggested that CD44s are required for human ovarian cancer cell adhesion to the mesothelial cell surface of HA [58]. HA increases the adhesion of CD44-expressing ovarian cancer cells to peritoneal cells *in vitro* [59]. In recent years, the notion of so-called cancer stem cells has been popularized and many have postulated that the CSC may be responsible for metastasis [60]. CD44 surface expression is a marker for ovarian cancer stem cells [61], further suggesting a potential role(s) of CD44 in metastatic behavior.

EZH2 is a recently identified, key regulator of tumor angiogenesis [62]. The increase in endothelial EZH2 is a direct result of VEGF stimulation and indicates the presence of a paracrine circuit promoting angiogenesis. Ezh2 silencing in the tumor-associated endothelial cells using chitosan-packaged siRNA significantly inhibits tumor growth in an orthotopic ovarian cancer model. Ezh2 silencing in tumor endothelial cells results in decreased angiogenesis mediated by increased levels of the angiogenesis inhibitor, VASH1. Combined, these data provide a significant conceptual advance in our understanding of the regulation of angiogenesis in ovarian carcinoma, and support the potential for targeting ezh2 as a therapeutic approach.

The extracellular matrix (ECM) is composed of collagen, laminin, and fibronectin. The ECM provides structural support and functions as a physical barrier. In order to invade and metastasize, tumor cells need to destroy the basement membrane and other components of the ECM. Tumor cells produce proteolytic enzymes and regulate ECM degradation. Matrix metalloproteases (MMP) are the main group of proteolytic enzymes that are involved in tumor invasion and metastasis in cancer [63]. High expression levels of certain MMPs are related to the tumor invasion capacity. This has been shown in ovarian carcinoma with MMP-2 and MMP-9 expression [64]. In addition to extracellular matrix remodeling, MMPs are also critical in angiogenesis.

Angiogenesis is required to sustain tumor growth [65]. Without neovascularization, the tumor mass is restricted to within a tissue-diffusion distance of approximately 0.2 mm. Tumor vessels are recruited by sprouting from pre-existing vessels, in which interstitial tissue columns are inserted into the lumen of pre-existing vessels and partition the vessel lumen. MMPs are essential regulators during various phases of the angiogenic process, from the deposition and breakdown of the basement membrane of vascular structures to endothelial cell proliferation and migration. Since MMPs influence multiple critical processes in metastasis, they are an ideal target for treatment. Results in clinical trials with MMP inhibitors, however, have been unsuccessful [66,67]. A greater understanding of MMPs is required in order to refine the therapeutic inhibitors targeting them.

## 7. Conclusion

We described in detail the molecular regulation of ovarian cancer metastasis with a focus on the EMT and tumor microenvironment. We addressed metastasis suppressor genes, EMT, and the tumor microenvironment, in the context of ovarian cancer metastasis. Suppressing tumor metastasis is a promising means by which to reduce ovarian cancer mortality and remains an attractive area for future drug development.

## Figures and Tables

**Table 1:** Summary of ovarian cancer related metastatic suppressor genes and proposed mechanisms.

Reporter/Year/References	Metastatic suppressor genes	Proposed mechanisms and fuctions
Youn/2008/[3]	*Nm23*	Inhibition of ras signaling, cell migration
Prentice/2007/[4]	*KISS1* (*kisspeptins*)	Ligand for G-protein coupled receptor, Maintains dormancy at secondary sites
Hata/2007/[5]	*KISS1R*	G-protein coupled receptor, cell colonization
Houle/2002/[6]; Ruseva/2009/[7]	*KAI1*	Interacts with endothelial DARC to induce apoptosis, cell invasion
Kim/2012/[8]; Ho/2010/[9]	*E-cadherin*	Cell: cell interactions, EMT, cell invasion
Ren/2011/[10]	*OGR1*	GPCR signaling Cell migration, cell colonization
Zhang/2006/[11]; Sheng/2012/[12]	*BRMS1*	Transcriptional regulation via interaction with SIN3:HDAC complexes; down-regulates Ptdlns (4,5)P2 Cell colonization
Yeasmin/2011/[13]; Nakayama/2006/[14]	*MKK4*	Stress-activated MAPK signaling, EMT, cell invasion
